# Association of interleukin-6, ferritin, and lactate dehydrogenase with venous thromboembolism in COVID-19: a systematic review and meta-analysis

**DOI:** 10.1186/s12879-024-09205-3

**Published:** 2024-03-16

**Authors:** Haiyu Liu, Ningjing Guo, Qixian Zheng, Qianyuan Zhang, Jinghan Chen, Yuanyuan Cai, Qiong Luo, Qian Xu, Xiangqi Chen, Sheng Yang, Suyun Zhang

**Affiliations:** 1 Department of Pulmonary and Critical Care Medicine, Fujian Medical University Union Hospital, Fuzhou, Fujian 350001 P.R. China; 2 Department of Oncology Medicine, Fujian Medical University Union Hospital, Fuzhou, Fujian 350001 P.R. China; 3 Department of General Medicine, Fujian Medical University Union Hospital, Fuzhou, Fujian 350001 P.R. China; 4 Fujian Key Laboratory of Translational Research in Cancer and Neurodegenerative Diseases, Fuzhou, Fujian 350001 P.R. China; 5 Department of Internal Medicine, Fujian Medical University Union Hospital, Fuzhou, Fujian 350001 P.R. China

**Keywords:** VTE, COVID-19, Ferritin, IL-6, LDH, Cytokine storm, Thrombosis prevention and anticoagulation therapy

## Abstract

**Background:**

Coronavirus disease 2019 (COVID-19) is frequntly accompanied by venous thromboembolism (VTE), and its mechanism may be related to the abnormal inflammation and immune status of COVID-19 patients. It has been proved that interleukin-6 (IL-6), ferritin and lactate dehydrogenase (LDH) may play an important role in the occurrence of VTE in COVID-19 infection. But whether they can server as predictors for VTE in COVID-19 is still unclear. In this study, we performed a systematic review and meta-analysis to compare IL-6, ferritin and LDH in VTE and non-VTE COVID-19 patients in order to shed light on the prevention and treatment of VTE.

**Methods:**

Related literatures were searched in PubMed, Embase, Web of Science, Google Scholar, China National Knowledge Infrastructure (CNKI), WANGFANG. COVID-19 patients were divided into VTE group and non-VTE group. Meta-analysis was then conducted to compare levels of IL-6, ferritin and LDH between the two groups.

**Results:**

We finally included and analyzed 17 literatures from January 2019 to October 2022. There was a total of 7,035 COVID-19 patients, with a weighted mean age of 60.01 years. Males accounted for 62.64% and 61.34% patients were in intensive care unit (ICU). Weighted mean difference (WMD) of IL-6, ferritin and LDH was 31.15 (95% CI: 9.82, 52.49), 257.02 (95% CI: 51.70, 462.33) and 41.79 (95% CI: -19.38, 102.96), respectively. The above results indicated that than compared with non-VTE group, VTE group had significantly higher levels of IL-6 and ferritin but similar LDH.

**Conclusion:**

This systematic review and meta-analysis pointed out that elevated levels of IL-6 and ferritin were significantly possitive associated with VTE, thus could be used as biological predictive indicators of VTE among COVID-19 patients. However, no association was found between level of LDH and VTE. Therefore, close monitoring of changes in IL-6 and ferritin concentrations is of great value in assisting clinicans to rapidly identify thrombotic complications among COVID-19 patients, hence facilitating the timely effective managment. Further studies are required in terms of the clinical role of cytokines in the occurrence of VTE among COVID-19 infection, with more reliable systematic controls and interventional trials.

**Supplementary Information:**

The online version contains supplementary material available at 10.1186/s12879-024-09205-3.

## Background

Coronavirus disease 2019 (COVID-19) refers to a highly infectious respiratory disease caused by severe acute respiratory disease coronavirus 2 (SARS-CoV-2). Since December 2019, COVID-19 has rapidly developed into a global pandemic characterized by human-to-human transmission and rapid pathogenesis, leading to large-scale spread and death around the world [1]. COVID-19 remains a global crisis and requires the joint efforts of all mankind. As severe COVID-19 patients are frequntly accompanied by vascular endothelial injury and hypercoagulable state of blood, they will be more vulnerable to venous thromboembolism (VTE), which can result in adverse clinical outcomes if not taken seriously.

As an uncontrolled and dysfunctional immune response, cytokine storm shares similar immunopathogenesis in severe influenza and COVID-19, that is, releasing large amount of inflammatory cytokines such as tumor necrosis factor alpha (TNF-α), interleukin-6 (IL-6), IL-12, and IL-18, thereby causing potential acute respiratory distress syndrome (ARDS) and systemic organ failure. Current evidence has showed that with the deterioration of the disease, there will be increasing levels of ferritin, D-dimer, lactate dehydrogenase (LDH), and IL-6, suggesting higher risk of mortality [2–4].

Laboratory examination combined with clinical assessment can quickly analyze and comprehensively assess the patient's condition, as well as accurately guide clinicians to get the key points of diagnosis and treatment of COVID-19, thus carry out the best treatment strategy. IL-6, ferritin and LDH are worth further exploration due to their potential diagnostic and prognostic roles. By screening literatures of COVID-19 patients with VTE, this meta-analysis study particularly focused on the elevated levels of IL-6, ferritin and LDH, in order to provide better guide in clinical practice and shed light on the prevention and treatment of VTE among COVID-19 patients.

## Methods

### Study design

We conducted a systematic review and meta-analysis on levels of IL-6, ferritin, and LDH among patients diagnosed with COVID-19. Eligible This systematic review and meta-analysis has been reported abiding to the Cochrane Handbook [5] and the guidance from Preferred Reporting Items of Systematic Reviews and Meta-Analyses (PRISMA) checklist [6]. The study protocol has been registered on the International Prospective Register of Systematic Reviews (PROSPERO) (CRD42023388470).

### Search strategy

We designed a high-sensitivity search strategy that combining word clusters of IL-6, ferritin, and LDH with free-text and keyword synonym clusters of COVID-19 and VTE. A systemic literature search was performed on PubMed, Embase, Web of Science, Google Scholar, China National Knowledge Infrastructure (CNKI), and WANGFANG. We further searched with keywords “interleukin-6”, “ferritin”, “LDH”, and “COVID-19” on bioRxiv (http://www.biorxiv.org) server, medRxiv (http://www.biorxiv.org) server and Chinaxiv (http://biotech.chinaxiv.org) server, in order to identify potential pre-publication manuscripts that met the eligibility criteria. All literatures were from January 2019 to October 2022, and reference lists of all included articles were also reviewed for potential citation eligibility.

### Study selection and data extraction

Two reviewers independently screened literatures by title and abstract, and then reviewed full-text. Literatures were included if they described two or more COVID-19 patients with or without VTE and reported measurements of cytokine levels (especially IL-6, ferritin, and LDH). The primary outcomes are IL-6 and ferritin, while secondary outcome is LDH.

Inclusion criteria were as follows: (1) available results of comparison between VTE and non-VTE in COVID-19 infection; (2) available laboratory results of levels of LDH, IL-6 and ferritin. Literatures were excluded if (1) they were non-human studies or non-comparative studies; (2) COVID-19 was not the target disease; (3) no available data to extract; (5) non-original studies (e.g., letters, reviews, editorials).

Data were extracted from text, tables, and graphs in literatures, with a standardized data extraction form. Besides, we also collected study design and setup, age characteristics, gender characteristics, disease characteristics, ICU admission rate, etc. Discrepancies were resolved through discussion.

### Statistical analysis

Continuous variables (i.e., IL-6, ferritin and LDH) were expressed as mean and standard deviation (SD) or median and interquartile range (IQR). If IL-6, ferritin and LDH were presented as median and IQR in the original articles, we used data conversion method and data conversion tool website (https://www.math.hkbu.edu.hk/~tongt/papers/median2mean.html) [7, 8].

Numerical differences between VTE group and non-VTE group were expressed as weighted mean difference (WMD) and 95% confidence interval (CI) for continuous variables, and odds ratio (OR) and 95% CI for categorical variables. Median and IQR of continuous variables were converted to mean and SD according to the method described by Wan et al. [8]. Heterogeneity was quantified using the I^2^ statistic and *P* value. I^2^ > 50% or *P* < 0.05 denoted considerable heterogeneity, and a random effects model was used to combine the results, otherwise, a fixed effects model was used. The threshold for significance was set at *P* < 0.05 [5]. All statistical analyses were conducted using Review Manager, Version 5.4 (Cochrane Collaboration, Oxford, UK) and STATA, Version 12.0 (StataCorp LP, College Station, Texas).

### Literature quality evaluation

To assess the quality of the included literatures, we used the Newcastle–Ottawa Scale (NOS) (https://www.ohri.ca/programs/CIinical_epidemiology/oxford.asp). The NOS scale used in this study had a total score of 9 (Table 2), and literature with a score of 7–9 was considered to be of excellent quality; literature with a score of 5–6 was considered to be of good quality; and literature with a score of less than 5 was considered to be of poor quality and was excluded from the follow-up study.

## Results

**Fig. 1:**
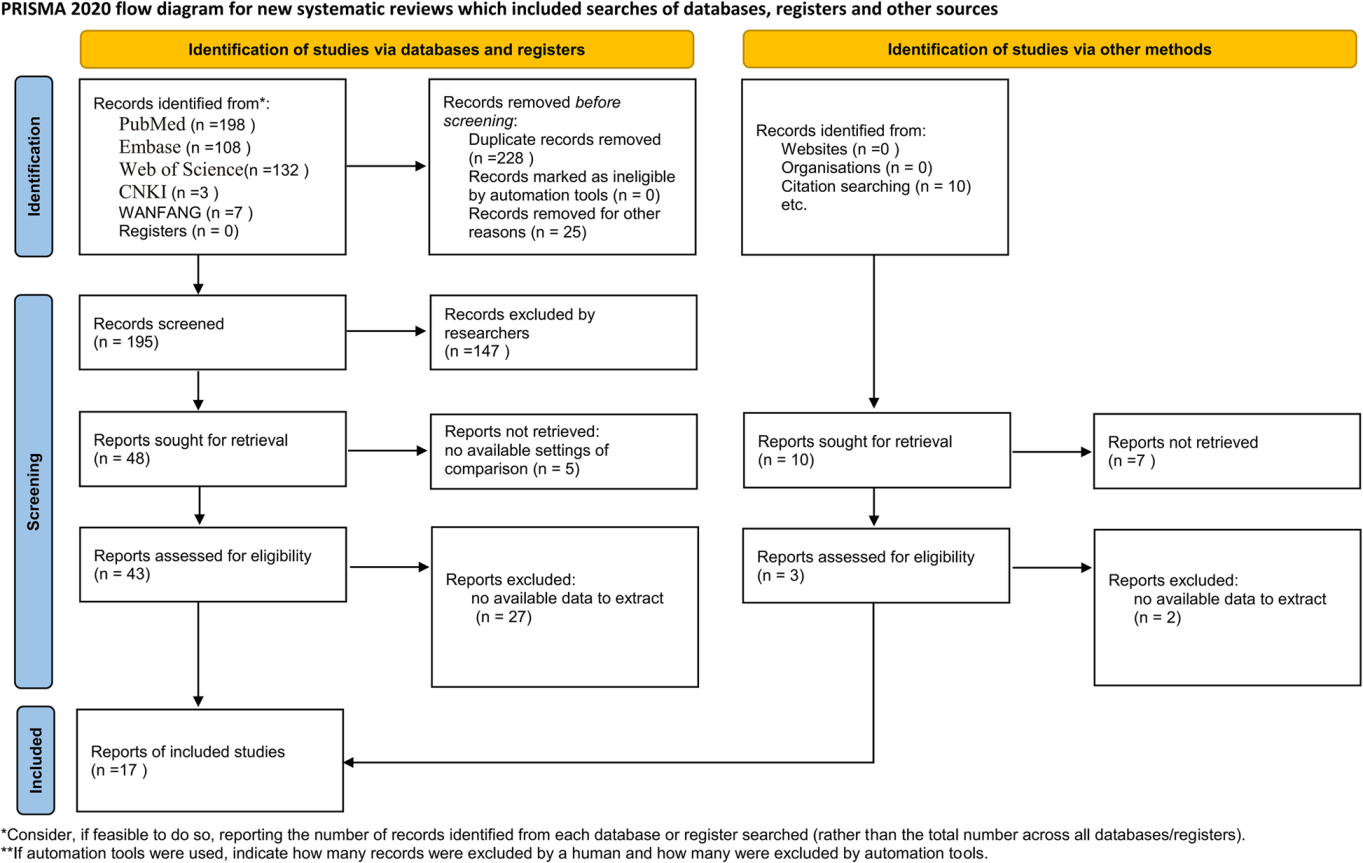
Flowchart for article inclusion

As shown in Fig. 1, a total of 208 studies were obtained with our search strategy, after excluding repeated articles and screening title and abstract, we retained 51 eligible studies. Further, 34 studies were excluded after full-text screening, and we finally had 17 literatures [9–25] for meta-analysis, including 12 retrospective cohort studies and 5 prospective cohort studies. The main characteristics of these studies were displayed in Table 1. The results of meta-analysis were the combined effects of IL-6, ferritin, and LDH in VTE group and non-VTE group.

**Table 1:**
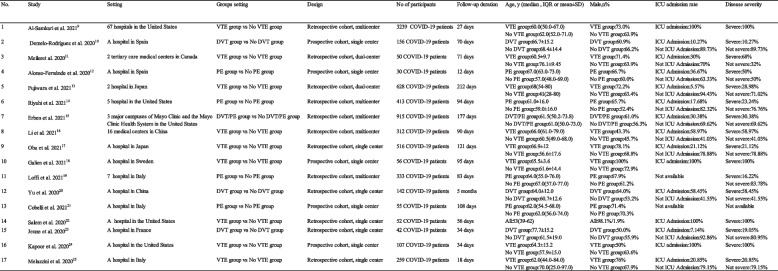
Table of basic information about the included articles

*VTE* Venous Thromboembolism, *DVT* Deep Venous Thrombosis, *PE* Pulmonary Embolism

Disease severity was defined as (i) need for ICU intensive care (ii) need for mechanical ventilation with tracheal intubation (iii) severe lung invasion as demonstrated by CT imaging (iv) acute respiratory failure (v) Severe and or Critical according to the WHO COVID-19 Severity Classification.

The presence of one of the above symptoms was defined as severe, and the absence of any of the above symptoms was defined as non-severe.

There was a total of 7,035 COVID-19 patients, with a weighted mean age of 60.01 years. Males accounted for 62.64% and 61.34% patients were in intensive care unit (ICU). The sample size ranged from 30 to 3,239, and the follow-up time ranged from 12 to 212 days.

**Fig. 2:**
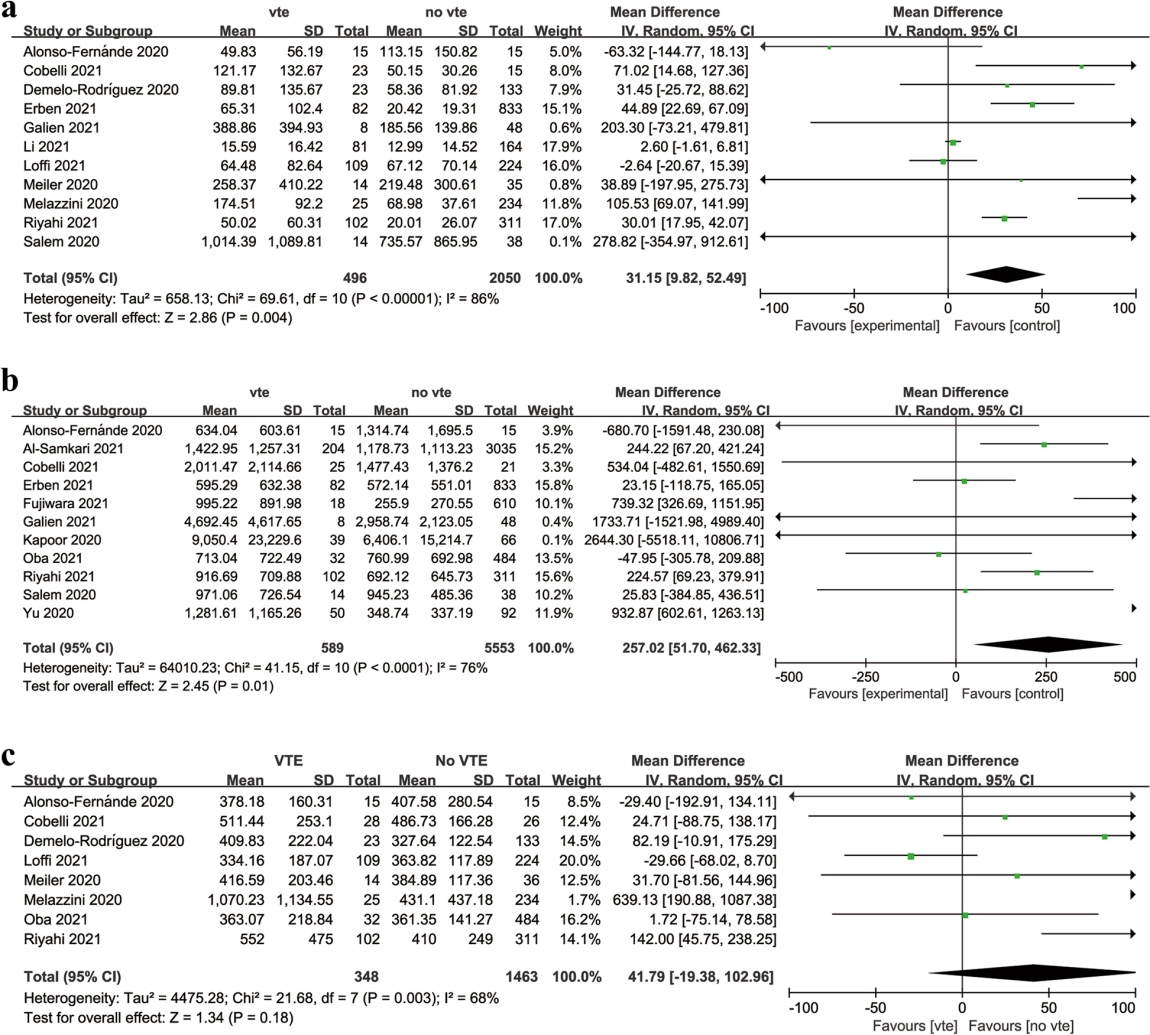
Forest plots of the correlation between IL-6, ferritin, LDH and the occurrence of venous thromboembolism in COVID-19 patients. **A** Forest plot of IL-6. **B** Forest plot of ferritin. Forest plot of LDH

Forest plots of the three studies were shown in Fig. 2. For levels of IL-6, ferritin and LDH, the random effect model (I-V heterogeneity, Cohen's d) was conducted. The results were as follows: (1) IL-6: I^2^ = 86%, *P* = 0. 004,WMD = 31.15 (95% CI: 9.82, 52.49); (2) ferritin: I^2^ = 76%, *P* = 0.01,WMD = 257.02 (95% CI: 51.70, 462.33); (3) LDH: I^2^ = 68%, *P* = 0.18,WMD = 41.79 (95% CI: -19.38, 102.96).

**Fig. 3:**
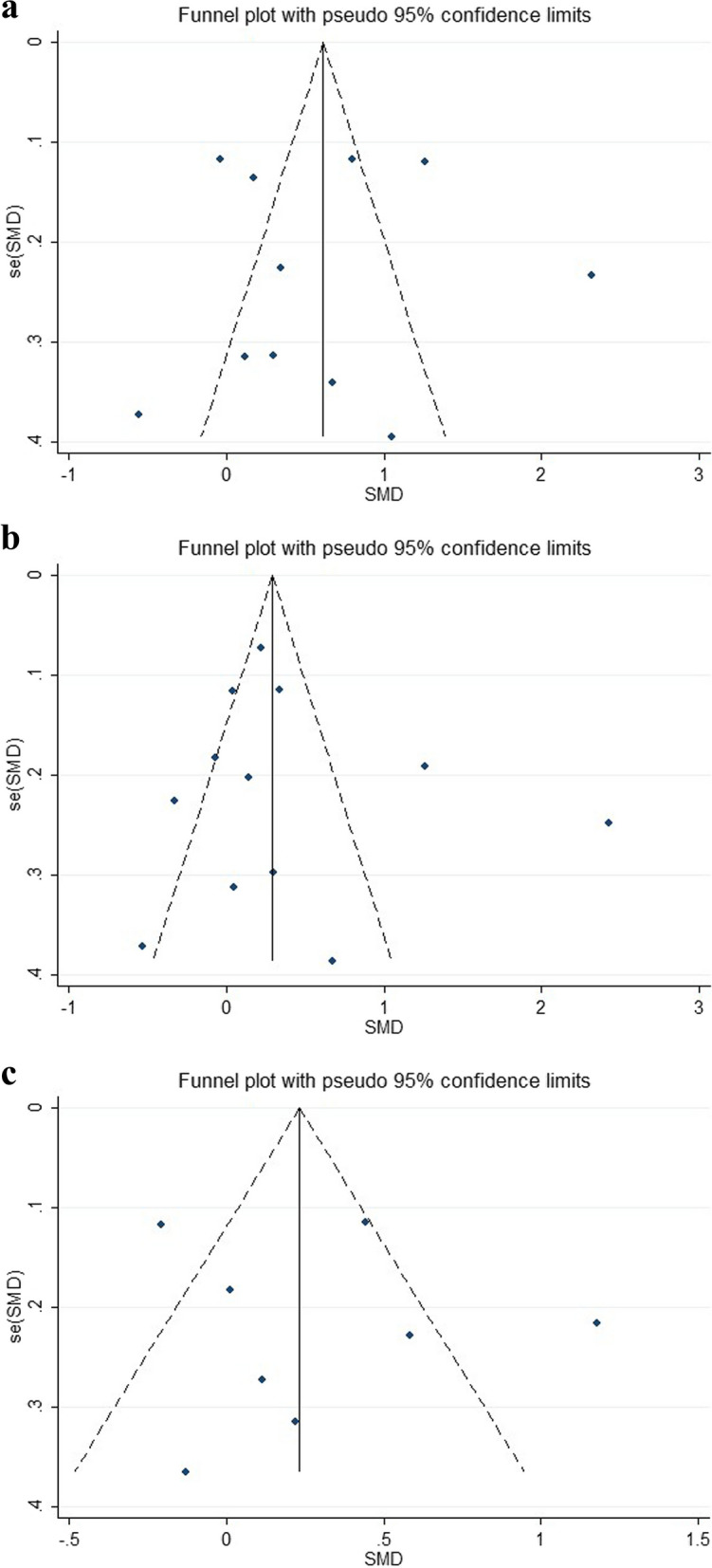
Funnel plots of the correlation between IL-6, ferritin, and LDH and the development of venous thromboembolism in COVID-19 patients. **A** Funnel plot of IL-6. **B** Funnel plot of ferritin. **C** Funnel plot of LDH

**Fig. 4:**
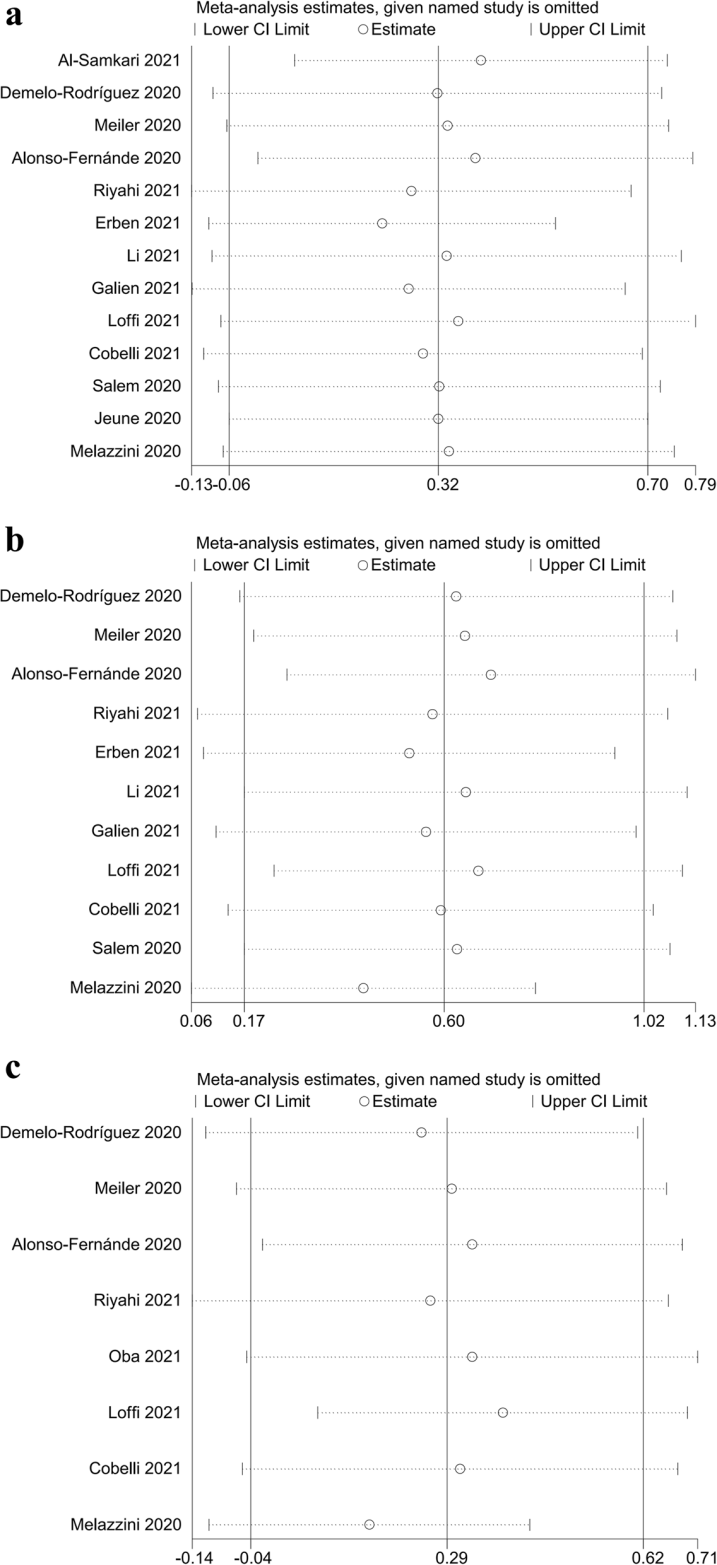
Sensitivity analysis of the correlation between IL-6, ferritin, and LDH and the development of venous thromboembolism in COVID-19 patients. **A** Sensitivity analysis of IL-6. **B** Sensitivity analysis of ferritin. **C** Sensitivity analysis of LDH

Since I^2^ of the above three forest plots were all greater than 50%, we then developed funnel plot and sensitivity analysis (Figs. 3 and  4) and the results were as follows: (1) IL-6: Alonso-Fernánde 2020, Melazzini 2020, Loffi 2021, Li 2021, and Erben 2021 had factors that might affect the results; (2) ferritin: Alonso-Fernánde 2020, Al-Samkari 2021 and Fujiwara 2021 had factors that might affect the results; (3) LDH: Melazzini 2020 and Loffi 2021had factors that might affect the results.

In addition, we also developed NOS for the evaluation of literature quality (Table 2), which showed that the selected articles were of good quality.

**Table 2:**
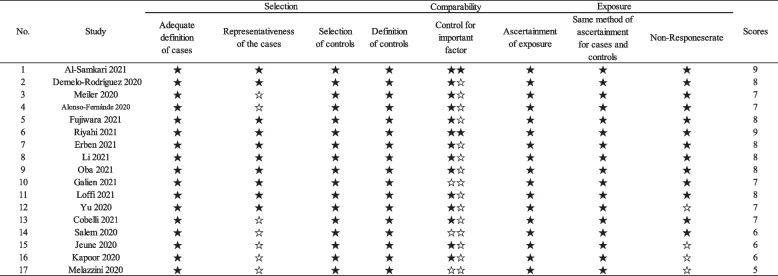
NOS scale for literature quality assessment

Based on the above analysis, we further conducted subgroup analysis to clarify the source of heterogeneity in terms of age (> 65 vs. ≤ 65 years), study duration (> 90 vs. ≤ 90 days), VTE type (deep venous thrombosis vs. pulmonary embolism), study design (retrospective cohort vs. prospective cohort), and ICU admission rate (100% vs. non-100%). Subgroup analysis (Table 3) suggested that the heterogeneity might be due to the large age span of study population, inconsistent disease severity, and retrospective study design.

**Table 3:**
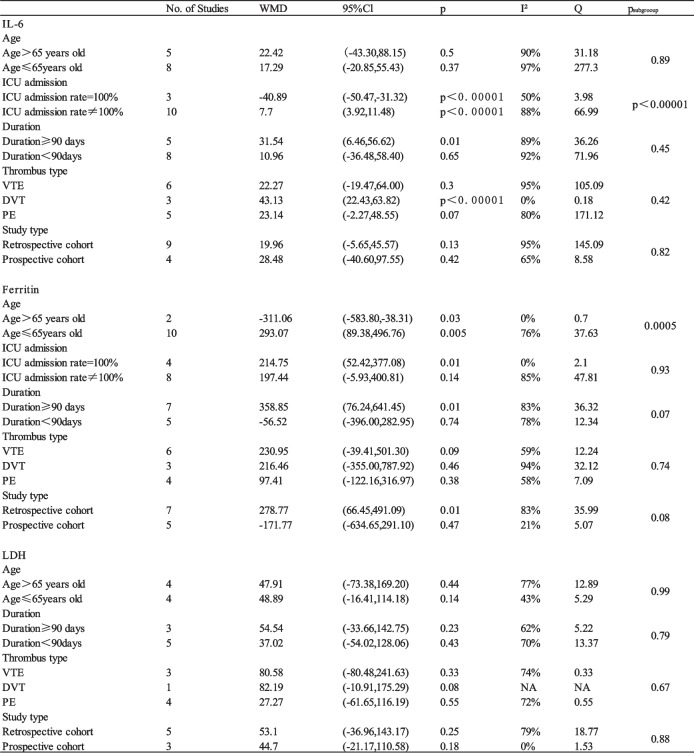
Table of subgroup analyses of initial results

*VTE* Venous Ehromboembolism, *DVT* Deep Venous Thrombosis, *PE* Pulmonary Embolism, *NA* Not available, *WMD* Weighted Mean Difference

## Discussion

The latest study has proved that compared with other viral pneumonia, COVID-19 infection has a higher risk of thromboembolism, which may be due to endothelial damage, cell membrane destruction and inactivity caused by the virus [26]. There is no doubt that with the greater cytokine storm and inflammation, thromboembolic complications are associated with severe and critical COVID-19. However, VTE and COVID-19 share many common vital signs and symptoms, it is difficult to identify in the early stage of COVID-19 complicated with VTE, which has brought challenges to clinical practice. Considering the high morbidity and mortality of severe COVID-19 combined with thromboembolism, more and more hospitals have systematically developed active anticoagulation protocols that are more comprehensive than standard VTE prophylaxis [27]. It remains an urgent need in clinical settings to identify potential biomarkers, timely assess the risk of VTE and guide the use of appropriate anticoagulation programs for thrombosis prevention, so as to reduce disease burden, improve the prognosis, thereby enhancing the quality of life of patients. This meta-analysis compared clinical commonly used cytokines (i.e., IL-6, ferritin and LDH) between COVID-19 patients with and without VTE, in order to investigate laboratory indicators for early monitoring of COVID-19 with VTE, as well as shed light on the prevention of VTE and clinical anticoagulation regimen. To our knowledge, this is the first meta-analysis focusing on the roles of IL-6, ferritin and LDH in the occurrence of VTE among COVID-19 infection.

Our results revealed that WMD of IL-6, ferritin and LDH was 31.15 (95% CI: 9.82, 52.49, *P* = 0.004), 257.02 (95% CI: 51.70, 462.33, *P* = 0.01) and 41.79 (95% CI: -19.38, 102.96, *P* = 0.18), respectively. The above results indicated that than compared with non-VTE group, VTE group had significantly higher levels of IL-6 and ferritin. Such significant difference was not found in LDH level, which might be related to the small number of studies included, but it is still worthy of continued attention.

Although levels of IL-6, ferritin and LDH can be higher in COVID-19 patients, their associations with VTE require further exploration. Previous meta-analysis has suggested that IL-6 level significantly elevates in COVID-19 patients and is associated with adverse clinical outcomes [28]. The underlying molecular mechanism may be that SARS-CoV-2 can stimulate monocytes/macrophages, to produce high levels of pro-inflammatory mediators such as IL-6 and IL-1β, which enhance cell death and lead to cytokine storm. IL-6 is a key inflammatory marker involved in the cytokine storm. Moreover, excessive IL-6 signal transduction can cause multiple organ injury through T cell maturation, vascular endothelial growth factor (VEGF) expression, increased vascular permeability, and decreased myocardial contractility [29]. Meanwhile, IL-6 monoclonal antibodies such as tocilizumab are considered as a potential biologic therapy to help restore the immune dysregulation associated with SARS-CoV-2. On the other hand, IL-6 is also related to the formation of VTE, which has been proved by some studies as an independent risk of VTE [30, 31]. The underlying mechanism is that IL-6-induced acute inflammation affects clotting factor levels, which will subsequently further modulate VTE. Besides, IL-6 genetic polymorphisms may contribute to VTE recurrence. Therefore, IL-6 plays an important role in the pathogenesis of VTE in COVID-19 patients, its excessive signal transduction can promote the development of the coagulation cascade and VTE. In addition, for COVID-19 patients with elevated IL-6 levels, timely administration of tocilizumab may improve cytokine release syndrome and reduce the risk of disseminated intravascular coagulation (DIC) [29].

Nowadays, the role of IL-6 in COVID-19 patients with VTE has drawn more and more attention. In 2020, Melazzini et al. first reported that compared with COVID-19 patients without VTE, the increased inflammatory indicators and serum proinflammatory cytokines, especially IL-6, should not be overlooked among those with VTE [25]. Riyahi et al. found in 2021 that COVID-19 patients with pulmonary embolism (PE) had higher IL-6 (*P* = 0.02) than those without PE [14]. In the same year, Sakr et al. indicated that that patients with COVID-19 and VTE were accompanied by increased IL-6 levels with disease progression [32]. At the same time, the elevated IL-6 may further result in adverse events. Erben et al. pointed out in 2021 that higher IL-6 levels in patients with COVID-19 and VTE were associated with both higher risks of ICU admission and rehabilitation after discharge [15]. However, some researchers hold different opinions. In 2021, Nurmohamed et al. demonstrated that there was significant relation between growing lipoprotein (a) and incidence of VTE in COVID-19 patients [33]. Since they also reported that the increased lipoprotein (a) was positively correlated with IL-6 (*r* = 0.44, 95% CI: 0.30–0.56, *P* < 0.001), elevated IL-6 not associated with venous thromboembolism, it provided a new idea for us to explore the role of IL-6 in the progression of VTE in patients with COVID-19 that elevated lipoprotein (a) is a potential cause of venous thromboembolism and elevated IL-6.

Our results further supported an positive association between elevated IL-6 levels and VTE among patients with COVID-19. The results will be helpful for clinicians to monitor the level of IL-6, to determine the risk of VTE, to apply appropriate VTE prevention regimen, as well as to guide the clinical use of IL-6 monoclonal antibodies.

Elevated ferritin levels due to cytokine storm and secondary hemophagocytic lymphohistiocytosis (sHLH) have also been previously reported in patients with critical COVID-19 [34]. In the meantime, the elevated ferritin can further affect the occurrence of adverse events. Alwafi et al. in 2020 conducted multivariate logistic regression analysis and found that mortality in COVID-19 patients was higher when ferritin level was greater than 400mcg/L [35]. Elevated serum ferritin is a feature of HLH who is well-known as a complication of viral infection that is strongly associated with poor prognosis in COVID-19 patients, and patients with impaired lung disease are more likely to have increased ferritin levels [2, 36]. It is worth mentioning that a meta-analysis revealed that COVID-19 patients with thrombotic complications had higher ferritin level than those without thrombotic complications, suggesting that there was a hyperinflammatory state in thrombotic patients [37]. Also, ferritin is not only the result of excessive inflammation, but plays a pathogenic role in the inflammatory process by binding to T cell immunoglobulin and mucin domain containing molecules-2 (TIM-2) to promote the expression of a variety of proinflammatory mediators. In addition, the H chain of ferritin is able to activate macrophages to secrete inflammatory cytokines [38]. High ferritin concentrations contribute to cytokine release in SARS-CoV-2 infection, promoting a hypercoagulable state [39].

In 2020, Pizzi et al. first identified that compared with non-COVID-19 patients, COVID-19 patients had significantly higher ferritin [40], followed by Oba et al. who clarified in 2021 that elevated ferritin was a useful biomarker for thrombosis in patients with COVID-19 [17]. Also in 2021, Sakr et al. [32] and Riyahi et al. [14] reported that there was an increasing ferritin level in COVID-19 patients with VTE and PE. On the contrary, by using Cox regression analysis, in 2020, Moll et al. implied that there was no significant correlation between ferritin level and increased risk of VTE among COVID-19 patients in ICU [41]. However, as the study population was limited to ICU admission, there should be some research bias from the perspective of patient selection.

Our results further supported an positive association between elevated ferritin levels and VTE in patients with COVID-19, which might be helpful for clinicians to detect the deterioration of disease, to have continuous vigilance of VTE by monitoring the ferritin level, to implement appropriate thromboprophylaxis protocols, to guide the use of plasma exchange/high-volume hemofiltration/dexamethasone to reduce the ferritin level, and thus to improve the clinical prognosis.

Currently, elevated LDH have been widely reported in COVID-19 cases, especially critical cases. As valuable biomarkers, LDH and IL-6 levels are positively correlated with disease severity, thus are effective in surveillance of critical COVID-19 patients. In addition, elevated baseline LDH levels are significantly associated with ARDS and mortality. It is important to note that LDH in non-critical cases decreased gradually within 10 days after admission, while LDH in critical or fatal cases did not decrease significantly throughout the course of the disease. Both IL-6 and LDH are independent predictive laboratory values for assessing COVID-19 severity, and early declines may be associated with better outcomes. Because higher levels of LDH are observed in nonsurvivors in the early stages of disease, measuring it at admission than during the ICU can have greater predictive value [2, 42, 43]. Therefore, LDH is considered as a valuable biomarker in severe and critical COVID-19 patients, especially those with cardiac comorbidities.

At present, the relationship between elevated LDH and venous thrombosis in patients with COVID-19 remains unclear. In 2020, Melazzini et al. first reported that COVID-19 patients with VTE had statistically higher LDH (*P* = 0.04) than those without VTE, pointing out that increased inflammatory indicators and serum proinflammatory cytokine levels should lead to clinical suspicion of VTE [25]. In the same year, Chen et al. conducted univariate analysis and concluded that elevated LDH levels in patients with COVID-19 might be associated with thrombosis, and that levels were significantly higher in VTE group than in non-VTE group [44]. Pizzi et al. also confirmed that LDH levels were significantly higher in COVID-19 patients compared to non-COVID-19 patients [39]. Compared with COVID-19 patients not complicated with PE, Riyahi et al. demonstrated that those who had PE presented elevated LDH (*P* = 0.001) in 2021 [14]. In the same year, Kumar et al. revealed that higher levels of LDH were associated with a higher incidence of VTE among COVID-19 patients. LDH mainly exists in human tissue cells, and its increase may be related to intravascular erythrocyte hemolysis and tissue cell damage. When VTE occurs, because of blood circulation disorders, there will be local tissue ischemia, hypoxia, edema and degeneration, and even necrosis. LDH in tissue cells is released into the blood, resulting in increased LDH levels [45].

Meanwhile, the elevated LDH can further affect the occurrence of adverse events. In 2022, Prince et al. found an increased risk of bleeding complications in hospitalized COVID-19 patients treated for VTE, especially in catheterized patients with elevated LDH [46]. Therefore, the use of low-molecular-weight heparin for the prevention and treatment of VTE in hospitalized COVID-19 patients with elevated LDH should be considered in light of the increased risk of bleeding complications during the treatment. Before starting anticoagulant therapy, clinicians should check and continuously monitor serum fibrinogen and LDH in order to reduce bleeding complications [45]. However, in 2022, Jurin et al. confirmed that VTE was independently associated with lower LDH levels in a multivariate logistics model [47].

Our results indicated no significant association between LDH and VTE in COVID-19 patients, which was contrary to previous findings and our hypothesis. This might be due to the limited controlled trials (*n* = 8) on the relation between LDH and COVID-19 with or without VTE, also, the included studies might havesome heterogeneity. In addition, COVID-19 disease is usually associated with high LDH levels and is associated with critical illness [48, 49]. In this study, we also found that LDH was usually elevated in both vte and non-vte groups and was higher than 300 U/L, which may be a major potential reason for the statistically non-significant difference in LDH between the two groups. Since current researchers believe that elevated LDH levels tend to be associated with VTE in COVID-19 patients, more reliable clinical studies are needed to further explore the underlying causes.

To sum up, there remains heterogeneity and controversy among the clinical research on the roles of IL-6, ferritin and LDH in VTE in COVID-19 patients, our study revealed that elevated levels of IL-6 and ferritin were significantly positive associated with VTE, while no association was found between level of LDH and VTE in COVID-19 infections. Therefore, close monitoring of changes in IL-6 and ferritin concentrations in COVID-19 patients, especially those at high risk for VTE, is of great value in determining changes in the disease and in reducing the rates of severe illness, VTE morbidity and mortality. Serial measurements of IL-6 and ferritin can predict impending asymptomatic thrombopoiesis and other critical clinical manifestations in SARS-CoV-2-infected patients. At the same time, we cannot ignore the important role played by LDH in the critical transformation of COVID-19 disease, which needs to be closely monitored on an ongoing basis. Different health service levels, types of virus strains, types and dose of vaccines, availability of thromboprophylaxis, and thromboprophylaxis regimens may lead to different results of clinical trials. Therefore, our results only provided a preliminary conclusion for reference, which still needs verification in the future.

There are some limitations in this study. First, incidence of VTE might be underestimated in our COVID-19 patients, because incidence of thrombotic events diagnosed on the basis of routine clinical carewas often less than that seen on computed tomography pulmonary angiography (CTPA). Second, this study only considered impact of the three biomarkers alone on VTE, but the combination of two or three indicators could have considerable clinical value. Further reliable studies are required for supplements. Third, the purpose of this meta-analysis was to explore the correlation between concentrations of three cytokines, IL-6, ferritin and LDH, and the occurrence of VTE in patients with COVID-19, and thus the patients included were varying in ages and severity in order to observe and analyze the problem in a wider, more detailed and deeper way, and therefore it was unavoidable that some degree of heterogeneity appeared. Our team addressed the problem of high I² by first identifying the studies that influenced the results by funnel plot and sensitivity analysis, including Alonso-Fernánde 2020, Melazzini 2020, Loffi 2021, Li 2021, Erben 2021, Al-Samkari 2021, Fujiwara 2021. And it was found that 1 of the above articles was a prospective study and the rest were retrospective and included COVID-19 patients with a wide age range and varying disease severity. Further subgroup analysis of this paper also verified that the potential factors leading to high heterogeneity could be retrospective studies, age, and disease severity; Of course, if the team could have included prospective studies in the same age group or with similar disease severity, meta-analysis would have allowed for a more multidimensional view and enriched the results of the study; In addition, the best way to address high heterogeneity may be to include more reliable studies in the future to more accurately identify the sources of heterogeneity, we will follow up with the appropriate studies in the future, and further explore the effects of factors such as type of study, age, disease severity, regional factors, ethnicity, gender composition, and strain differences, etc., on the relationship between the occurrence of VTE in patients with COVID-19. Finally, although this meta-analysis mainly explored the relationship between cytokines and VTE from the perspective of inflammation, inflammation is closely related to immunity. Previous studies documented that in vitro blockade of PD-1 could almost normalize the immune expression of IL-1β, IL-1RA and IL-8 levels among patients with COVID-19 and restore T cell function, thus recovered the immune abnormalities to normal [50]. This also provides us with potential ideas to explore the relationship between cellular immune factors and VTE in COVID-19 patients, which still needs further exploration.

## Conclusion

This systematic review and meta-analysis pointed out that elevated levels of IL-6 and ferritin were significantly positive associated with VTE, thus could be used as biological predictive indicators of VTE and provided an emerging research direction in the field of VTE prevention among COVID-19 patients. However, no association was found between level of LDH and VTE in COVID-19 infections. Therefore, close monitoring of changes in IL-6 and ferritin concentrations is of great value in assisting clinicans to rapidly identify thrombotic complications among COVID-19 patients, hence facilitating the timely effective managment. Further studies are required in terms of the clinical role of cytokines in the occurrence of VTE among COVID-19 infection, with more reliable systematic controls and interventional trials.

### Supplementary Information

**Supplementary Material 1.**

**Supplementary Material 2.**

## Data Availability

All data generated or analysed during this study are included in this published article and its supplementary files. The data that support the findings of this study are available from the corresponding author upon reasonable request.
