# Supplementary material for: Association of interleukin-6, ferritin, and lactate dehydrogenase with venous thromboembolism in COVID-19: a systematic review and meta-analysis
Source: BMC Infect Dis. 2024 Mar 16;24:324. doi: 10.1186/s12879-024-09205-3 (PMC10943892; doi:10.1186/s12879-024-09205-3)
Supplement: Supplementary file 2 — Supplementary Material 2. [file 12879_2024_9205_MOESM2_ESM.docx]

**Supplementary Materials: Association of Interleukin-6, Ferritin, and Lactate Dehydrogenase with Venous Thromboembolism in COVID-19: A Systematic Review and Meta-analysis**

**File: Search Strategy**

**PUBMED**

***Covid-19 (#1)***

(((COVID-19[MeSH Terms]) OR (((((((((((((((((((((((((((((((((((((COVID 19[Title/Abstract]) OR (2019-nCoV Infection[Title/Abstract])) OR (2019 nCoV Infection[Title/Abstract])) OR (2019-nCoV Infections[Title/Abstract])) OR (Infection, 2019-nCoV[Title/Abstract])) OR (SARS-CoV-2 Infection[Title/Abstract])) OR (Infection, SARS-CoV-2[Title/Abstract])) OR (SARS CoV 2 Infection[Title/Abstract])) OR (SARS-CoV-2 Infections[Title/Abstract])) OR (2019 Novel Coronavirus Disease[Title/Abstract])) OR (2019 Novel Coronavirus Infection[Title/Abstract])) OR (COVID-19 Virus Infection[Title/Abstract])) OR (COVID 19 Virus Infection[Title/Abstract])) OR (COVID-19 Virus Infections[Title/Abstract])) OR (Infection, COVID-19 Virus[Title/Abstract])) OR (Virus Infection, COVID-19[Title/Abstract])) OR (COVID19[Title/Abstract])) OR (Coronavirus Disease 2019[Title/Abstract])) OR (Disease 2019, Coronavirus[Title/Abstract])) OR (Coronavirus Disease-19[Title/Abstract])) OR (Coronavirus Disease 19[Title/Abstract])) OR (Severe Acute Respiratory Syndrome[Title/Abstract])) OR (Coronavirus 2 Infection[Title/Abstract])) OR (COVID-19 Virus Disease[Title/Abstract])) OR (COVID 19 Virus Disease[Title/Abstract])) OR (COVID-19 Virus Diseases[Title/Abstract])) OR (Disease, COVID-19 Virus[Title/Abstract])) OR (Virus Disease, COVID-19[Title/Abstract])) OR (SARS Coronavirus 2 Infection[Title/Abstract])) OR (2019-nCoV Disease[Title/Abstract])) OR (2019 nCoV Disease[Title/Abstract])) OR (2019-nCoV Diseases[Title/Abstract])) OR (Disease, 2019-nCoV[Title/Abstract])) OR (COVID-19 Pandemic[Title/Abstract])) OR (COVID 19 Pandemic[Title/Abstract])) OR (Pandemic, COVID-19[Title/Abstract])) OR (COVID-19 Pandemics[Title/Abstract]))) OR (SARS-CoV-2[MeSH Terms])) OR (((((((((((((((((((((((((SARS-CoV-2 Virus[Title/Abstract]) OR (SARS CoV 2 Virus[Title/Abstract])) OR (SARS-CoV-2 Viruses[Title/Abstract])) OR (Virus, SARS-CoV-2[Title/Abstract])) OR (2019 Novel Coronavirus[Title/Abstract])) OR (2019 Novel Coronaviruses[Title/Abstract])) OR (Coronavirus, 2019 Novel[Title/Abstract])) OR (Novel Coronavirus, 2019[Title/Abstract])) OR (COVID-19 Virus[Title/Abstract])) OR (COVID 19 Virus[Title/Abstract])) OR (COVID-19 Viruses[Title/Abstract])) OR (Virus, COVID-19[Title/Abstract])) OR (Wuhan Coronavirus[Title/Abstract])) OR (Coronavirus, Wuhan[Title/Abstract])) OR (COVID19 Virus[Title/Abstract])) OR (COVID19 Viruses[Title/Abstract])) OR (Virus, COVID19[Title/Abstract])) OR (Viruses, COVID19[Title/Abstract])) OR (Coronavirus Disease 2019 Virus[Title/Abstract])) OR (Severe Acute Respiratory Syndrome[Title/Abstract])) OR (Coronavirus 2[Title/Abstract])) OR (SARS Coronavirus 2[Title/Abstract])) OR (Coronavirus 2, SARS[Title/Abstract])) OR (2019-nCoV[Title/Abstract])) OR (Wuhan Seafood Market Pneumonia Virus[Title/Abstract]))

***vte(#2)***

((((("Venous Thromboembolism"[Mesh]) OR ((Venous Thromboembolism[Title/Abstract]) OR (Thromboembolism, Venous[Title/Abstract]))) OR (Venous Thrombosis[MeSH Terms])) OR (((((((((((((((((((((((((Phlebothrombosis[Title/Abstract]) OR (Phlebothromboses[Title/Abstract])) OR (Thrombosis, Venous[Title/Abstract])) OR (Thromboses, Venous[Title/Abstract])) OR (Venous Thromboses[Title/Abstract])) OR (Deep Vein Thrombosis[Title/Abstract])) OR (Deep Vein Thromboses[Title/Abstract])) OR (Thromboses, Deep Vein[Title/Abstract])) OR (Vein Thromboses, Deep[Title/Abstract])) OR (Vein Thrombosis, Deep[Title/Abstract])) OR (Deep-Venous Thrombosis[Title/Abstract])) OR (Deep-Venous Thromboses[Title/Abstract])) OR (Thromboses, Deep-Venous[Title/Abstract])) OR (Thrombosis, Deep-Venous[Title/Abstract])) OR (Deep-Vein Thrombosis[Title/Abstract])) OR (Deep-Vein Thromboses[Title/Abstract])) OR (Thromboses, Deep-Vein[Title/Abstract])) OR (Thrombosis, Deep-Vein[Title/Abstract])) OR (Thrombosis, Deep Vein[Title/Abstract])) OR (Deep Venous Thrombosis[Title/Abstract])) OR (Deep Venous Thromboses[Title/Abstract])) OR (Thromboses, Deep Venous[Title/Abstract])) OR (Thrombosis, Deep Venous[Title/Abstract])) OR (Venous Thromboses, Deep[Title/Abstract])) OR (Venous Thrombosis, Deep[Title/Abstract]))) OR (Pulmonary Embolism[MeSH Terms])) OR (((((((Pulmonary Embolisms[Title/Abstract]) OR (Embolism, Pulmonary[Title/Abstract])) OR (Embolisms, Pulmonary[Title/Abstract])) OR (Pulmonary Thromboembolisms[Title/Abstract])) OR (Pulmonary Thromboembolism[Title/Abstract])) OR (Thromboembolism, Pulmonary[Title/Abstract])) OR (Thromboembolisms, Pulmonary[Title/Abstract]))

***IL-6(#3)***

(Interleukin-6[MeSH Terms]) OR ((((((((((((((((((((((((((((((Interleukin 6[Title/Abstract]) OR (IL6[Title/Abstract])) OR (B-Cell Stimulatory Factor 2[Title/Abstract])) OR (B-Cell Stimulatory Factor-2[Title/Abstract])) OR (Differentiation Factor-2, B-Cell[Title/Abstract])) OR (Differentiation Factor 2, B Cell[Title/Abstract])) OR (B-Cell Differentiation Factor-2[Title/Abstract])) OR (B Cell Differentiation Factor 2[Title/Abstract])) OR (BSF-2[Title/Abstract])) OR (Hybridoma Growth Factor[Title/Abstract])) OR (Growth Factor, Hybridoma[Title/Abstract])) OR (IFN-beta 2[Title/Abstract])) OR (Plasmacytoma Growth Factor[Title/Abstract])) OR (Growth Factor, Plasmacytoma[Title/Abstract])) OR (Hepatocyte-Stimulating Factor[Title/Abstract])) OR (Hepatocyte Stimulating Factor[Title/Abstract])) OR (MGI-2[Title/Abstract])) OR (Myeloid Differentiation-Inducing Protein[Title/Abstract])) OR (Differentiation-Inducing Protein, Myeloid[Title/Abstract])) OR (Myeloid Differentiation Inducing Protein[Title/Abstract])) OR (B-Cell Differentiation Factor[Title/Abstract])) OR (B Cell Differentiation Factor[Title/Abstract])) OR (Differentiation Factor, B-Cell[Title/Abstract])) OR (Differentiation Factor, B Cell[Title/Abstract])) OR (IL-6[Title/Abstract])) OR (Interferon beta-2[Title/Abstract])) OR (Interferon beta 2[Title/Abstract])) OR (beta-2, Interferon[Title/Abstract])) OR (B Cell Stimulatory Factor-2[Title/Abstract])) OR (B Cell Stimulatory Factor 2[Title/Abstract]))

***Ferritin(#4)***

(Ferritins[MeSH Terms]) OR ((((Ferritin[Title/Abstract]) OR (Isoferritin[Title/Abstract])) OR (Basic Isoferritin[Title/Abstract])) OR (Isoferritin, Basic[Title/Abstract]))

***LDH(#5)***

(L-Lactate Dehydrogenase[MeSH Terms]) OR ((((Dehydrogenase, L-Lactate[Title/Abstract]) OR (L Lactate Dehydrogenase[Title/Abstract])) OR (Lactate Dehydrogenase[Title/Abstract])) OR (Dehydrogenase, Lactate[Title/Abstract]))

***Search Formula***

#1 AND #2 AND (#3 OR #4 OR #5)

**WEB OF SCIENCE**

*Query #1*

ALL=(“COVID-19” OR“COVID 19” OR“2019-nCoV Infection” OR“2019 nCoV Infection”OR“2019-nCoV Infections”OR“Infection, 2019-nCoV”OR“SARS-CoV-2 Infection”OR“Infection, SARS-CoV-2”OR“SARS CoV 2 Infection”OR“SARS-CoV-2 Infections”OR“2019 Novel Coronavirus Disease”OR“2019 Novel Coronavirus Infection”OR“COVID-19 Virus Infection”OR“COVID 19 Virus Infection”OR“COVID-19 Virus Infections”OR“Infection, COVID-19 Virus”OR“Virus Infection, COVID-19”OR“COVID19”OR“Coronavirus Disease 2019”OR“Disease 2019, Coronavirus”OR“Coronavirus Disease-19”OR“Coronavirus Disease 19”OR“Severe Acute Respiratory Syndrome”OR“Coronavirus 2 Infection”OR“COVID-19 Virus Disease”OR“COVID 19 Virus Disease”OR“COVID-19 Virus Diseases”OR“Disease, COVID-19 Virus”OR“Virus Disease, COVID-19”OR“SARS Coronavirus 2 Infection”OR“2019-nCoV Disease”OR“2019 nCoV Disease”OR“2019-nCoV Diseases”OR“Disease, 2019-nCoV”OR“COVID-19 Pandemic”OR“COVID 19 Pandemic”OR“Pandemic, COVID-19”OR“COVID-19 Pandemics”OR“SARS-CoV-2”OR“SARS-CoV-2 Virus”OR“SARS CoV 2 Virus”OR“SARS-CoV-2 Viruses”OR“Virus, SARS-CoV-2”OR“2019 Novel Coronavirus”OR“2019 Novel Coronaviruses”OR“Coronavirus, 2019 Novel”OR“Novel Coronavirus, 2019”OR“COVID-19 Virus”OR“COVID 19 Virus”OR“COVID-19 Viruses”OR“Virus, COVID-19”OR“Wuhan Coronavirus”OR“Coronavirus, Wuhan”OR“COVID19 Virus”OR“COVID19 Viruses”OR“Virus, COVID19”OR“Viruses, COVID19”OR“Coronavirus Disease 2019 Virus”OR“Severe Acute Respiratory Syndrome”OR“Coronavirus 2”OR“SARS Coronavirus 2”OR“Coronavirus 2, SARS”OR“2019-nCoV”OR“Wuhan Seafood Market Pneumonia Virus”)

*Query #2*

ALL=(“Venous Thromboembolism”OR“Thromboembolism, Venous”OR“Venous Thrombosis”OR“Phlebothrombosis”OR“Phlebothromboses”OR“Thrombosis, Venous”OR“Thromboses, Venous”OR“Venous Thromboses”OR“Deep Vein Thrombosis”OR“Deep Vein Thromboses”OR“Thromboses, Deep Vein”OR“Vein Thromboses, Deep”OR“Vein Thrombosis, Deep”OR“Deep-Venous Thrombosis”OR“Deep-Venous Thromboses”OR“Thromboses, Deep-Venous”OR“Thrombosis, Deep-Venous”OR“Deep-Vein Thrombosis”OR“Deep-Vein Thromboses”OR“Thromboses, Deep-Vein”OR“Thrombosis, Deep-Vein”OR“Thrombosis, Deep Vein”OR“Deep Venous Thrombosis”OR“Deep Venous Thromboses”OR“Thromboses, Deep Venous”OR“Thrombosis, Deep Venous”OR“Venous Thromboses, Deep”OR“Venous Thrombosis, Deep”OR“Pulmonary Embolism”OR“Pulmonary Embolisms”OR“Embolism, Pulmonary”OR“Embolisms, Pulmonary”OR“Pulmonary Thromboembolisms”OR“Pulmonary Thromboembolism”OR“Thromboembolism, Pulmonary”OR“Thromboembolisms, Pulmonary”)

*Query #3*

ALL=(“Interleukin-6”OR“Interleukin 6”OR“IL6”OR“B-Cell Stimulatory Factor 2”OR“B-Cell Stimulatory Factor-2”OR“Differentiation Factor-2, B-Cell”OR“Differentiation Factor 2, B Cell”OR“B-Cell Differentiation Factor-2”OR“B Cell Differentiation Factor 2”OR“BSF-2”OR“Hybridoma Growth Factor”OR“Growth Factor, Hybridoma”OR“IFN-beta 2”OR“Plasmacytoma Growth Factor”OR“Growth Factor, Plasmacytoma”OR“Hepatocyte-Stimulating Factor”OR“Hepatocyte Stimulating Factor”OR“MGI-2”OR“Myeloid Differentiation-Inducing Protein”OR“Differentiation-Inducing Protein, Myeloid”OR“Myeloid Differentiation Inducing Protein”OR“B-Cell Differentiation Factor”OR“B Cell Differentiation Factor”OR“Differentiation Factor, B-Cell”OR“Differentiation Factor, B Cell”OR“IL-6”OR“Interferon beta-2”OR“Interferon beta 2”OR“beta-2, Interferon”OR“B Cell Stimulatory Factor-2”OR“B Cell Stimulatory Factor 2”)

*Query #4*

ALL=(“Ferritins”OR“Ferritin”OR“Isoferritin”OR“Basic Isoferritin”OR“Isoferritin, Basic”)

*Query #5*

ALL=(“L-Lactate Dehydrogenase”OR“Dehydrogenase, L-Lactate”OR“L Lactate Dehydrogenase”OR“Lactate Dehydrogenase”OR“Dehydrogenase, Lactate” )

***Search Formula***

#1 AND #2 AND (#3 OR #4 OR #5)

**EMBASE**

*Query Results*

*#7* #1 AND #1 AND #6

*#6* #5 OR #4 OR #3

#5 (' L-Lactate Dehydrogenase':ab,ti OR ' Dehydrogenase, L-Lactate':ab,ti OR ' L Lactate Dehydrogenase':ab,ti OR ' Lactate Dehydrogenase':ab,ti OR ' Dehydrogenase, Lactate':ab,ti)

#4 (' Ferritins':ab,ti OR ' Ferritin':ab,ti OR ' Isoferritin':ab,ti OR ' Basic Isoferritin':ab,ti OR ' Isoferritin, Basic':ab,ti)

#3 (' Interleukin-6 ':ab,ti OR ' Interleukin 6 ':ab,ti OR ' IL6 ':ab,ti OR ' B-Cell Stimulatory Factor 2 ':ab,ti OR ' B-Cell Stimulatory Factor-2 ':ab,ti OR ' Differentiation Factor-2, B-Cell ':ab,ti OR ' Differentiation Factor 2, B Cell ':ab,ti OR ' B-Cell Differentiation Factor-2 ':ab,ti OR ' B Cell Differentiation Factor 2 ':ab,ti OR ' BSF-2 ':ab,ti OR ' Hybridoma Growth Factor ':ab,ti OR ' Growth Factor, Hybridoma ':ab,ti OR ' IFN-beta 2 ':ab,ti OR ' Plasmacytoma Growth Factor ':ab,ti OR ' Growth Factor, Plasmacytoma ':ab,ti OR ' Hepatocyte-Stimulating Factor ':ab,ti OR ' Hepatocyte Stimulating Factor ':ab,ti OR ' MGI-2 ':ab,ti OR ' Myeloid Differentiation-Inducing Protein ':ab,ti OR ' Differentiation-Inducing Protein, Myeloid ':ab,ti OR ' Myeloid Differentiation Inducing Protein ':ab,ti OR ' B-Cell Differentiation Factor ':ab,ti OR ' B Cell Differentiation Factor ':ab,ti OR ' Differentiation Factor, B-Cell ':ab,ti OR ' Differentiation Factor, B Cell':ab,ti OR ' IL-6':ab,ti OR ' Interferon beta-2':ab,ti OR ' Interferon beta 2':ab,ti OR ' beta-2, Interferon':ab,ti OR ' B Cell Stimulatory Factor-2':ab,ti OR ' B Cell Stimulatory Factor 2':ab,ti)

#2 ('Venous Thromboembolism ':ab,ti OR 'Thromboembolism, Venous':ab,ti OR 'Venous Thrombosis':ab,ti OR ' Phlebothrombosis ':ab,ti OR ' Phlebothromboses ':ab,ti OR ' Thrombosis, Venous ':ab,ti OR ' Thromboses, Venous ':ab,ti OR '':ab,ti OR ' Venous Thromboses ':ab,ti OR ' Deep Vein Thrombosis ':ab,ti OR ' Deep Vein Thromboses ':ab,ti OR ' Thromboses, Deep Vein ':ab,ti OR ' Vein Thromboses, Deep ':ab,ti OR ' Vein Thrombosis, Deep ':ab,ti OR ' Deep-Venous Thrombosis ':ab,ti OR ' Deep-Venous Thromboses ':ab,ti OR ' Thromboses, Deep-Venous ':ab,ti OR ' Thrombosis, Deep-Venous ':ab,ti OR ' Deep-Vein Thrombosis ':ab,ti OR ' Deep-Vein Thromboses ':ab,ti OR ' Thromboses, Deep-Vein ':ab,ti OR ' Thrombosis, Deep-Vein ':ab,ti OR ' Thrombosis, Deep Vein ':ab,ti OR ' Deep Venous Thrombosis ':ab,ti OR ' Deep Venous Thromboses ':ab,ti OR ' Thromboses, Deep Venous ':ab,ti OR ' Thrombosis, Deep Venous ':ab,ti OR ' Venous Thromboses, Deep ':ab,ti OR ' Venous Thrombosis, Deep ':ab,ti OR ' Pulmonary Embolism ':ab,ti OR ' Pulmonary Embolisms ':ab,ti OR ' Embolism, Pulmonary ':ab,ti OR ' Embolisms, Pulmonary ':ab,ti OR ' Pulmonary Thromboembolisms ':ab,ti OR ' Pulmonary Thromboembolism ':ab,ti OR ' Thromboembolism, Pulmonary ':ab,ti OR ' Thromboembolisms, Pulmonary ':ab,ti)

#1 ('COVID-19':ab,ti OR 'COVID 19':ab,ti OR '2019-nCoV Infection':ab,ti OR '2019 nCoV Infection':ab,ti OR '2019-nCoV Infections':ab,ti OR 'Infection, 2019-nCoV':ab,ti OR 'SARS-CoV-2 Infection':ab,ti OR ' Infection, SARS-CoV-2 ':ab,ti OR ' SARS CoV 2 Infection ':ab,ti OR ' SARS-CoV-2 Infections ':ab,ti OR ' 2019 Novel Coronavirus Disease ':ab,ti OR ' 2019 Novel Coronavirus Infection ':ab,ti OR ' COVID-19 Virus Infection ':ab,ti OR ' COVID 19 Virus Infection ':ab,ti OR ' COVID-19 Virus Infections ':ab,ti OR ' Infection, COVID-19 Virus ':ab,ti OR ' Virus Infection, COVID-19 ':ab,ti OR ' COVID19 ':ab,ti OR ' Coronavirus Disease 2019 ':ab,ti OR ' Disease 2019, Coronavirus ':ab,ti OR ' Coronavirus Disease-19 ':ab,ti OR ' Coronavirus Disease 19 ':ab,ti OR ' Severe Acute Respiratory Syndrome ':ab,ti OR ' Coronavirus 2 Infection ':ab,ti OR ' COVID-19 Virus Disease ':ab,ti OR '':ab,ti OR ' COVID 19 Virus Disease ':ab,ti OR ' COVID-19 Virus Diseases ':ab,ti OR 'Disease, COVID-19 Virus':ab,ti OR 'Virus Disease, COVID-19':ab,ti OR 'SARS Coronavirus 2 Infection':ab,ti OR '2019-nCoV Disease':ab,ti OR '2019 nCoV Disease':ab,ti OR '2019-nCoV Diseases':ab,ti OR 'Disease, 2019-nCoV':ab,ti OR 'COVID-19 Pandemic':ab,ti OR 'COVID 19 Pandemic':ab,ti OR 'Pandemic, COVID-19':ab,ti OR 'COVID-19 Pandemics':ab,ti OR 'SARS-CoV-2':ab,ti OR 'SARS-CoV-2 Virus':ab,ti OR 'SARS CoV 2 Virus':ab,ti OR 'SARS-CoV-2 Viruses':ab,ti OR 'Virus, SARS-CoV-2':ab,ti OR '2019 Novel Coronavirus':ab,ti OR '2019 Novel Coronaviruses':ab,ti OR 'Coronavirus, 2019 Novel':ab,ti OR 'Novel Coronavirus, 2019':ab,ti OR 'COVID-19 Virus':ab,ti OR 'COVID 19 Virus':ab,ti OR 'COVID-19 Viruses':ab,ti OR 'Virus, COVID-19':ab,ti OR 'Wuhan Coronavirus':ab,ti OR 'Coronavirus, Wuhan':ab,ti OR 'COVID19 Virus':ab,ti OR 'COVID19 Viruses':ab,ti OR 'Virus, COVID19':ab,ti OR 'Viruses, COVID19':ab,ti OR 'Coronavirus Disease 2019 Virus':ab,ti OR 'Severe Acute Respiratory Syndrome ':ab,ti OR 'Coronavirus 2':ab,ti OR 'SARS Coronavirus 2':ab,ti OR 'Coronavirus 2, SARS':ab,tiOR '2019-nCoV':ab,ti OR 'Wuhan Seafood Market Pneumonia Virus':ab,ti)

**Google Scholar, China National Knowledge Infrastructure (CNKI), WANGFANG, bioRxiv, medRxiv, Chinaxiv**

***Search Formula***

(COVID-19)AND(vte)AND((IL-6)OR(ferritin)OR(LDH))
